# PBMC transcriptomic signatures reflect immune dynamics and disease activity in psoriatic arthritis

**DOI:** 10.3389/fimmu.2026.1701395

**Published:** 2026-02-24

**Authors:** Ruihong Hou, Yang Liu, Dengfeng Xue, Ruonan Wu, Ke Xu, Liyun Zhang

**Affiliations:** 1 Department of Rheumatology and Immunology, Shanxi Bethune Hospital, Shanxi Academy of Medical Sciences, Third Hospital of Shanxi Medical University, Tongji Shanxi Hospital, Taiyuan, China; 2 Department of Breast Surgery, The Second Hospital of Shanxi Medical University, Taiyuan, China; 3 School of Public Health, Shanxi Medical University, Taiyuan, China

**Keywords:** arthritis, immunity, PBMCs, psoriatic, signatures, transcriptional

## Abstract

**Objective:**

To characterize systemic transcriptomic alterations across psoriatic arthritis (PsA) disease states, including distinctions from psoriasis-only (PsO), signatures of disease activity, and treatment-responsive changes in peripheral blood mononuclear cells.

**Methods:**

RNA sequencing was performed in patients with PsA, psoriasis without arthritis (PSO) and healthy controls (HC). Four analytical comparisons were examined: PsA versus healthy controls, PsA versus PsO, active versus remission PsA, and paired pre- versus post-treatment PsA. Differential gene expression(DEGs), functional enrichment, and protein-protein interaction analyses were integrated to delineate immune and metabolic programs across conditions.

**Results:**

PsA showed extensive transcriptional alterations relative to healthy individuals, characterized by activation of adaptive immune pathways, cytokine signaling, and coordinated metabolic adjustments. Compared with PsO, PsA exhibited pronounced dysregulation of extracellular matrix components, platelet activation, coagulation, and complement pathways, indicating systemic involvement not observed in skin-limited disease. Disease activity was associated with enhanced angiogenesis, cell adhesion, cell adhesion and migration, and extracellular matrix remodeling, alongside enrichment of PI3K-Akt, MAPK, IL-17, and complement/coagulation signaling. Paired longitudinal profiling demonstrated substantial transcriptomic reversibility after treatment, including attenuation of immune, stromal, and vascular signatures. Across analyses, recurrent patterns emerged: pervasive peripheral immune activation, distinct vascular and hemostatic alterations differentiating PsA from PsO, and consistent metabolic remodeling with partial normalization following therapy.

**Conclusion:**

This multi-dimensional transcriptomic study delineates immune, vascular, and metabolic perturbations across PsA disease states and highlights their dynamic modulation with disease activity and treatment. These findings provide an integrated framework for understanding systemic inflammatory patterns in PsA and support future mechanistic and translational investigation.

## 1 Introduction

Psoriatic arthritis (PsA) is a chronic immune-mediated inflammatory disease affecting the joints and entheses, commonly coexisting with cutaneous psoriasis (1). Beyond musculoskeletal manifestations, PsA is increasingly recognized as a systemic disorder associated with metabolic dysregulation, endothelial dysfunction, and elevated cardiovascular risk (2). Despite therapeutic advances, early diagnosis, patient stratification, and disease monitoring remain challenging (3), reflecting the heterogeneity of PsA and the lack of molecular markers capable of capturing its dynamic immunologic states.

Current models of PsA pathogenesis emphasize the roles of the IL-23/IL-17 axis (4, 5), activated T cells (6), antigen-presenting cells, and innate-adaptive immune crosstalk (7). Emerging evidence also implicates metabolic rewiring, stromal activation, and vascular abnormalities in disease development (8, 9) However, much of this knowledge (10–13) derives from studies of isolated tissues—such as synovium or skin—or from cross-sectional analyses limited to a single disease state. Although informative, such studies provide only a partial view of the systemic immune landscape that accompanies PsA progression and therapeutic response.

Peripheral blood mononuclear cells (PBMCs) are a practical and clinically accessible compartment that can reflect circulating immune perturbations (14, 15). Nevertheless, PBMC-based transcriptomic studies in PsA remain limited (12, 13), typically focusing on comparisons with healthy controls, with few analyses incorporating PsO as a comparator or examining disease activity and treatment-induced changes. As a result, it remains unclear how transcriptional programs differ between PsA and PsO, which pathways characterize active versus quiescent disease, and how systemic immune signatures evolve following therapy. These gaps limit our understanding of PsA heterogeneity and impede the development of molecular tools for disease assessment.

To address these limitations, we conducted an integrated transcriptomic analysis of PBMCs across multiple PsA disease states—active disease, remission, and paired pre- and post-treatment samples—and compared these profiles with those from PsO patients and healthy controls. Through differential expression, functional enrichment, and protein–protein interaction analyses, our study aims to delineate systemic immune and metabolic perturbations across the PsA spectrum and to characterize transcriptional signatures associated with disease activity and therapeutic modulation. This multi-dimensional framework provides a more comprehensive understanding of circulating inflammatory programs in PsA and advances insights into its systemic immunobiology.

## 2 Methods

### 2.1 Study participants and sample collection

Fourteen patients with PsA were recruited from the Department of Rheumatology at Shanxi Bethune Hospital, all fulfilling the classification criteria for psoriatic arthritis (CASPAR) (16). All PsA participants were newly diagnosed and treatment-naïve at the time of blood sampling. None had received immunosuppressive agents or biologic therapies prior to enrollment. Six patients had intermittently used non-steroidal anti-inflammatory drugs (NSAIDs) for symptomatic relief, while the remaining patients had not taken any anti-inflammatory or disease-modifying medications. For comparison, five age- and sex-matched patients with cutaneous psoriasis (PSO) (17) without clinical or imaging evidence of arthritis (confirmed by an experienced dermatologist), and five healthy controls (HC) were included. Exclusion criteria: Patients were excluded if they had any of the following: (1) current or past malignancy; (2) acute infections (e.g., respiratory, urinary, gastrointestinal) or chronic infections (e.g., tuberculosis, hepatitis B/C, HIV); (3) other autoimmune or inflammatory rheumatic diseases (e.g., rheumatoid arthritis, systemic lupus erythematosus, inflammatory bowel disease); (4) prior or current use of immunosuppressants, biologic agents, or systemic glucocorticoids; (5) severe hepatic or renal dysfunction; (6) uncontrolled metabolic or cardiovascular conditions likely to influence immune status; (7) pregnancy or lactation.

Disease activity was assessed using the disease activity index for psoriatic arthritis (DAPSA) (18). PsA patients were stratified into active (DAPSA > 4) and remission (DAPSA ≤ 4) subgroups (19, 20). The severity of cutaneous psoriasis was evaluated using the psoriasis area and severity index (PASI), with PASI score ≥3 defining moderate-to-severe disease. A subset of four PsA patients underwent longitudinal transcriptomic analysis before and after initiation of treatment with specify therapy [e.g. biologic agent]. All patients provided written informed consent, and the study was approved by the Ethics Committee of Shanxi Bethune Hospital (Approval No. LYLL-2024-004/PJ30).

### 2.2 RNA extraction, library preparation, and sequencing

Peripheral blood samples were collected into EDTA tubes. PBMCs were isolated via Ficoll-Paque density gradient centrifugation within 2 hours of collection, washed in phosphate-buffered saline (PBS), and stored in TRIzol reagent at −80 °C. Total RNA was extracted using TRIzol (Thermo, USA), and RNA quality was assessed with a NanoDrop spectrophotometer and Agilent 2100 Bioanalyzer. Samples meeting the criteria of A260/A280 between 1.8-2.1, A260/A230 >2.0, RNA integrity number (RIN) ≥7.0, and concentration >50 ng/Ml were included. Polyadenylated mRNA was enriched using oligo(dT) magnetic beads, fragmented, and reverse-transcribed into cDNA. Strand-specific libraries were constructed using dUTP incorporation and size-selected to ~300 bp inserts. Libraries were amplified by PCR and sequenced on an Illumina NovaSeq 6000 platform (paired-end, 2×150 bp).

### 2.3 Transcriptome data analysis

Adapters trimming and quality filtering were performed using Cutadapt. Clean reads were aligned to the GRCh38 human reference genome using HISAT2, and transcript assembly and quantification were carried out using StringTie. Low-abundance transcripts were removed prior to downstream analysis using a counts-per-million (CPM) cutoff of ≥1 in at least three samples to reduce background noise. After quality control filtering, 42,682 genes were retained for subsequent analysis. Gene-level read counts were imported into R and normalized using the trimmed mean of M-values (TMM) method, followed by voom transformation to model the mean-variance relationship and compute precision weights. Principal component analysis (PCA) was conducted on the normalized matrix to assess sample clustering and detect potential outliers. No outliers were identified, and the PCA plot is provided in **Supplementary Figure 1**. Differential expression analysis was performed using the limma package (v3.66.0) across four predefined comparisons: PsA *vs*. HC, PsA *vs*. PSO, active *vs*. remission PsA, and pre- *vs*. post-treatment PsA. For the comparisons between PsA and HC, and between PsA and PSO, analyses were adjusted for age, sex, and BMI, as these variables were available for all individuals in the respective groups and allowed for stable model fitting. Other metabolic comorbidities (e.g., hypertension, diabetes) were not included as covariates due to limited sample size and incomplete matching, which could compromise model robustness. This limitation is acknowledged in the Discussion. Differentially expressed genes (DEGs) were defined as those with |log2 fold change| > 1 and adjusted *p* value (FDR) < 0.05. Volcano plots now display adjusted *p*-values, with threshold lines corresponding to FDR < 0.05, and heatmaps and volcano plots were generated using ComplexHeatmap and ggplot2.

### 2.4 Functional enrichment and network analysis

Gene Ontology (GO), Kyoto Encyclopedia of Genes and Genomes (KEGG), and Gene Set Enrichment Analysis (GSEA) were performed using clusterProfiler (v4.6.2) to identify enriched biological processes and pathways. For GO and KEGG analyses, terms or pathways with a nominal *p*-value < 0.05 were considered significant. To account for multiple testing, FDR values were calculated using the Benjamini–Hochberg (BH) procedure, and FDR-adjusted values are reported in the **Supplementary Tables**. GSEA was performed using a preranked approach based on gene-level statistics. Gene sets were considered significantly enriched if they met all of the following criteria: an absolute normalized enrichment score (|NES|) > 1, nominal *p*-value < 0.05, and FDR q-value < 0.25. Multiple-testing correction for GSEA was performed using the BH method. To identify metabolism-related DEGs in the pre- *versus* post-treatment comparison, DEGs were functionally annotated using GO Biological Process terms and KEGG pathway databases. Genes annotated to metabolism-related GO terms or KEGG metabolic pathways were classified as metabolism-related DEGs. Protein-protein interaction (PPI) networks were constructed using the STRING database (v11.5) (**Supplementary Method 1**). Only DEGs that met the statistical thresholds (|log_2_FC| > 1 and FDR < 0.05) were included, and no first- or second-order neighbors were added, ensuring that the networks reflected interactions exclusively among the identified DEGs. Interactions were included based on a confidence score >0.4. For downstream analysis, STRING interaction tables were imported into Cytoscape (v3.9.1). Hub genes were identified using the Maximal Clique Centrality (MCC) algorithm implemented in the cytoHubba plugin. The MCODE algorithm was applied to detect densely connected subnetworks and to highlight potential functional modules within the DEG-derived PPI networks.

### 2.5 Quantitative real-time PCR validation

To experimentally validate the transcriptomic findings, an independent cohort consisting of peripheral blood samples from eight treatment-naïve PsA patients and eight HCs was used for quantitative real-time PCR (qRT-PCR). Total RNA was reverse-transcribed into cDNA, and qRT-PCR was conducted using SYBR Green chemistry on a real-time PCR system. Expression levels of LY96, S100A4, and CAPG were normalized to an internal GAPDH and calculated using the 2^−ΔΔCt method. All reactions were performed in technical triplicates. Differences between groups were assessed using Student’s t-test or Mann–Whitney U test, as appropriate, with a two-sided P < 0.05 considered statistically significant.

### 2.6 Statistical analysis

Comparisons of clinical characteristics were conducted using SPSS version 21.0. Data are presented as mean ± standard deviation (SD), median (interquartile range, IQR), or number (percentage), as appropriate. Continuous variables were compared using Student’s t test for normally distributed data or the Mann–Whitney U test for non-normally distributed data. Categorical variables, including sex, presence of nail involvement, low-titer ANA positive, HLA-B27 positive, and metabolic syndrome were compared using Fisher’s exact test due to small sample sizes. A two-tailed *p*-value < 0.05 was considered statistically significant.

## 3 Results

### 3.1 PsA patient characteristics

A total of 14 patients with PsA were enrolled, comprising 10 males and 4 females, with a mean age of 42.36 ± 8.94 years. All patients initially presented with cutaneous psoriasis, with a mean disease duration of 11.17 ± 7.03 years and a median duration of arthritis symptoms 4 years (IQR: 2–7). The median latency from skin to joint involvement was 4 years (IQR: 0–12.75). Nail involvement was observed in 7 patients (50.0%). Clinical phenotypes included oligoarthritis (n = 8, 57.14%), polyarthritis (n = 4, 28.57%), axial PsA (n = 1, 7.14%), and distal interphalangeal arthritis (n = 1, 7.14%). The mean body mass index (BMI) was 25.01 ± 2.29 kg/m², and 8 patients (57.14%) met the diagnostic criteria for metabolic syndrome. At the time of PBMC sampling, 9 patients (64.29%) were classified as having active PsA (PsAa), and 5 (35.71%) were in remission (PsAn), based on DAPSA scores. Moderate-to-severe cutaneous involvement was present in 5 patients (35.71%), while 9 (64.29%) had mild cutaneous disease. Detailed demographic and clinical characteristics are summarized in **Table 1**.

**Table 1:** Baseline clinical and demographic characteristics of the study population.

Variable	PsA (N=14)	PsO (N=5)	HC (N=5)	*P*_1_	*P*_2_
Age, years, (mean±SD)	42.36±8.94	38.40±9.45	42.60±13.78	0.414	0.964
Female,N(%)	4/14 (25.8%)	3/5 (60.0%)	3/5 (60.0%)	0.305	0.305
Height, cm, (mean±SD)	170.11±7.77	164.20±8.14	166.20±4.55	0.167	0.308
Weight, kg, median (IQR)	75.00(65.00,79.25)	65.00(58.00,88.00)	63.30(60.50,72.25)	0.815	0.103
BMI, kg/m², (mean±SD)	25.01±2.29	26.43±5.53	23.75±1.32	0.427	0.264
Duration of cutaneous psoriasis, years, (mean±SD)	11.17±7.03	12.12±10.27	–	0.821	–
Duration of arthritis symptoms, years, median (IQR)	4 (2,7)	–	–	–	–
Latency from skin to joint involvement, years, median (IQR)	4 (0,12.75)	–	–	–	–
DAPSA score, (mean ± SD)	17.02±15.06	–	–	–	–
> 4, N(%)	9/14 (64.3%)				
≤ 4, N(%)	5/14 (35.7%)				
PASI score, (mean±SD)	4.19±5.75	5.64±3.63	–	0.607	–
≥ 3, N(%)	5/14 (35.7%)	4/5 (80.0%)			
< 3, N(%)	9/14 (64.3%)	1/5 (20.0%)			
Patient global assessment, (mean±SD)	3.71±2.30	2.40±1.67	–	0.261	–
Pain VAS, (mean±SD)	3.79±2.39	–	–	–	–
Psoriatic nail changes, N(%)	7/14 (50.0%)	2/5 (40.0%)	–	1.000	–
Low-titer ANA positive, N(%)	3/14 (21.4%)	2/5 (40.0%)	–	0.570	–
HLA-B27 positive, N(%)	1/14 (7.1%)	0/5 (0)	–	1.000	–
ESR, mm/h, median (IQR)	5.00(3.50,11.50)	5.00(4.00,5.50)	–	0.850	–
CRP, mg/L, median (IQR)	6.18(2.00,21.71)	1.00(0.55,1.16)	–	0.002	–
Tender joint counts, (mean±SD)	5.36±7.06	–	–	–	–
Swollen joint counts, (mean±SD)	2.93±4.23	–	–	–	–
Metabolic syndrome ≥1 component, N(%)	8/14 (57.1%)	1/5 (20.0%)	0/5 (0)	0.303	–
Diabetes mellitus	3/14 (21.4%)	1/5 (20.0%)	0/5 (0)	–	–
Hypertension	5/14 (35.7%)	0/5 (0)	0/5 (0)	–	–
Cardiovascular and cerebrovascular diseases	0/14 (0)	0/5 (0)	0/5 (0)	–	
Gout	0/14 (0)	0/5 (0)	0/5 (0)	–	–
Hepatic steatosis	6/14 (42.9%)	0/5 (0)	0/5 (0)	–	–
PsA subtype, N(%)					
Oligoarthritis	8/14 (57.1%)	–	–	–	–
Polyarthritis	4/14 (28.6%)	–	–	–	–
Axial PsA	1/14 (7.1%)	–	–	–	–
Distal interphalangeal arthritis	1/14 (7.1%)	–	–	–	–
Treatment given, N(%)					
NSAIDs	9/14 (64.3%)	0/5 (0)	–	–	–
MTX	3/14 (21.4%)	0/5 (0)	–	–	–
bDMARDs					
TNF-α inhibitors	3/14 (21.4%)	0/5 (0)	–	–	–
IL-17 inhibitors	3/14 (21.4%)	2/5 (40.0%)	–	–	–
IL-23 inhibitors	1/14 (7.1%)	0/5 (0)	–	–	–
JAK inhibitors	2/14 (14.3%)	0/5 (0)	–	–	–

### 3.2 PsA vs healthy controls: inflammatory activation and metabolic remodeling

**Figure 1:**
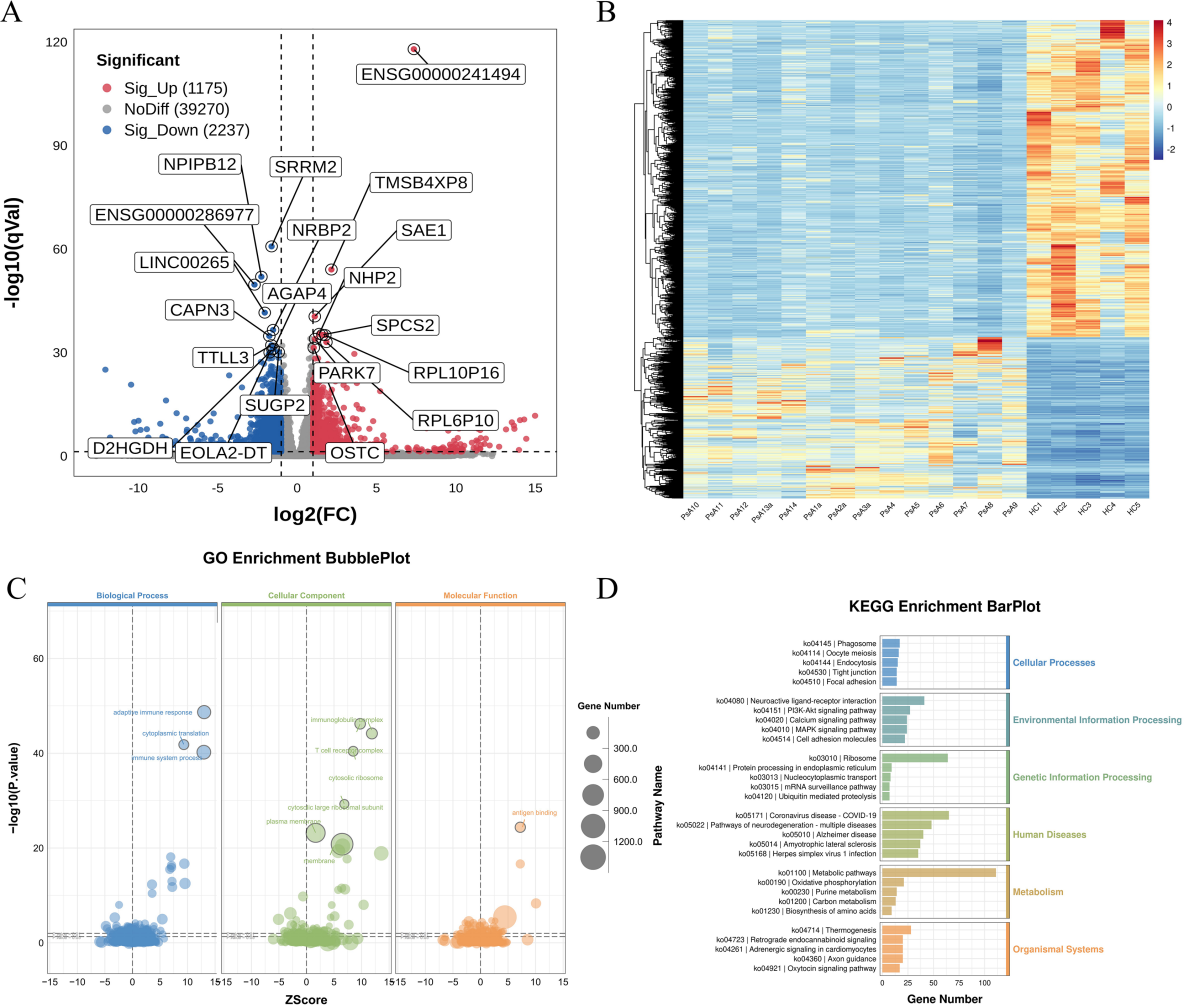
Differential transcriptomic profiling of PBMCs from PsA patients and HC. **(A)** Volcano plot showing DEGs between PsA (n=14) and healthy controls (HC, n=5). DEGs were defined as |log_2_FC| > 1 and FDR < 0.05. Upregulated and downregulated genes are shown in red and blue, respectively, with the most significantly dysregulated transcripts labeled. **(B)** Heatmap of the top 100 DEGs ranked by significance, illustrating clear separation between PsA and HC samples. Colors represent normalized expression levels. **(C)** GO enrichment analysis of DEGs, displaying significantly enriched Biological Process, Cellular Component, and Molecular Function terms. Bubble size corresponds to gene counts, and color reflects enrichment significance. **(D)** KEGG pathway enrichment bar plot. Pathways are grouped by functional category, and bar length indicates the number of DEGs mapped to each term. Key enriched pathways include metabolic programs, immune activation pathways, and signaling cascades associated with inflammatory responses. GO terms and KEGG pathways shown were selected based on nominal *p*-value < 0.05, with FDR values calculated using the Benjamini–Hochberg method. PBMCs, peripheral blood mononuclear cells; PsA, psoriatic arthritis; HC, healthy controls; DEGs, differentially expressed genes; GO, Gene Ontology; KEGG, Kyoto Encyclopedia of Genes and Genomes.

Differential expression analysis between 14 PsA patients and 5 HC identified 3412 DEGs, including 1175 upregulated and 2237 downregulated transcripts (**Supplementary Table 1**). The volcano plot (**Figure 1A**) illustrates the genome-wide distribution of DEGs, with log_2_ fold change on the x-axis and -log_10_ adjusted *p*-value on the y-axis. Strongly upregulated genes—including SAE1, TMSB4XP8, and NHP2—are positioned on the right, whereas markedly downregulated genes—such as SRRM2, NPIPB12, and CAPN3—cluster on the left. The corresponding heatmap (**Figure 1B**) illustrates the expression patterns across all samples, with columns representing individuals and rows representing genes, demonstrating a clear separation between PsA and HC profiles.

GO enrichment analysis (**Figure 1C**) highlights the biological processes, cellular components, and molecular functions most affected in PsA. Bubble sizes reflects the number of genes contributing to each term, and color intensity corresponds to statistical significance. Enriched biological processes were dominated by pathways related to adaptive immune activation, including antigen binding and T-cell receptor complex organization. Enriched cellular components included ribosomal subunits, immunoglobulin complexes, and T-cell receptor complexes, consistent with broad upregulation of immune effector activity.

KEGG pathway analysis (**Figure 1D**) provides an integrated overview of the top enriched pathways. Within the Metabolism functional category, metabolic pathways (ko01100) represented the largest gene set, accompanied by significant enrichment in oxidative phosphorylation (ko00190), purine metabolism (ko00230), carbon metabolism (ko01200), and biosynthesis of amino acids (ko01230), suggesting coordinated remodeling of metabolic and bioenergetic programs in PsA.

GSEA (**Supplementary Figure 2**) further confirmed activation of immune and metabolic signaling cascades. Positively enriched gene sets included T cell receptor signaling, Th17 differentiation, NF-κB, PI3K, MAPK, VEGF, and cytokine–cytokine receptor interaction pathways. Multiple metabolic pathways—such as unsaturated fatty acid metabolism, arginine and proline metabolism, cysteine and methionine metabolism, and sphingolipid pathways—showed significant enrichment, underscoring the interplay between immune activation and metabolic remodeling in PsA pathogenesis.

To explore external consistency, we compared our PsA *versus* HC DEGs with publicly available PBMC transcriptomic datasets. However, due to disease heterogeneity and differences in data preprocessing, only a limited number of genes overlapped across datasets (**Supplementary Figure 3**, **Supplementary Tables 2**-**8**), precluding robust cross-dataset validation. Among the eight genes consistently shared across all datasets, three representative genes—LY96, S100A4, and CAPG—were selected for quantitative qRT-PCR analysis based on their established relevance to PsA-related immune activation and their consistent differential expression in the RNA-seq dataset. qRT-PCR analysis in PBMCs demonstrated that LY96, S100A4, and CAPG were all significantly upregulated in PsA patients compared with HC (**Supplementary Figures 4A-C**). The direction and relative magnitude of expression changes were highly concordant with the transcriptomic results.

### 3.3 PsA vs psoriasis: distinct immune and coagulation signatures

**Figure 2:**
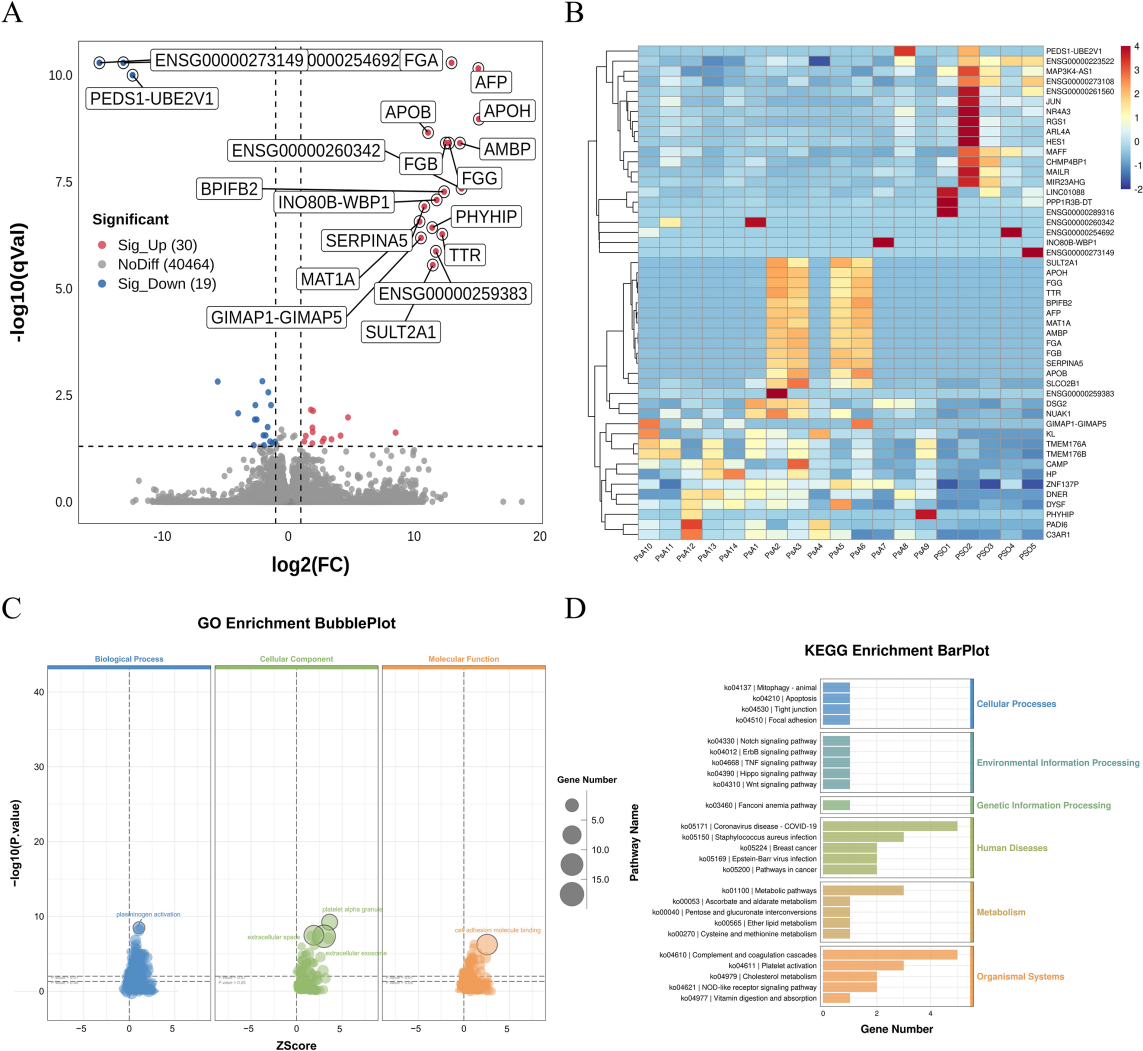
Differential transcriptomic profiling between PsA and PsO PBMCs. **(A)** Volcano plot showing DEGs between PsA (n=14) and PsO (n=5) PBMCs. Red dots indicate significantly upregulated genes (|log_2_FC| > 1, FDR < 0.05), blue dots indicate downregulated genes, and grey dots represent non-significant genes. Representative DEGs are labeled. Upregulated genes on the right side displayed similar log_2_ fold-change values but differed in statistical significance, leading to a vertical clustering pattern in the volcano plot. All samples were generated in a single sequencing batch and processed using identical quality control and normalization procedures. This pattern may reflect coordinated transcriptional upregulation of functionally related gene modules in active PsA. **(B)** Heatmap of DEGs showing distinct transcriptional patterns between PsA and PsO samples. Rows represent genes and columns represent individual patients. Color scale reflects normalized expression values (red: higher; blue: lower). **(C)** GO enrichment bubble plot of DEGs. Enriched Biological Process, Cellular Component, and Molecular Function terms are displayed. Dot size indicates the number of genes per term, and color corresponds to statistical significance. **(D)** KEGG pathway enrichment bar plot. Pathways are grouped by functional category, and bar length indicates the number of DEGs mapped to each term. Enriched pathways include complement and coagulation cascades, platelet activation, and amino acid metabolism. GO terms and KEGG pathways shown were selected based on nominal *p*-value < 0.05, with FDR values calculated using the Benjamini–Hochberg method. PBMCs, peripheral blood mononuclear cells; PsA, psoriatic arthritis; PsO, psoriasis; DEGs, differentially expressed genes; GO, Gene Ontology; KEGG, Kyoto Encyclopedia of Genes and Genomes.

Compared with PSO patients, PsA patients exhibited 30 upregulated and 19 downregulated genes (**Supplementary Table 9**). The volcano plot (**Figure 2A**) highlights prominent upregulated transcripts, including FGA, APOB, APOH, FGB, AMBP, and INO80B-WBP1, which cluster on the right side of the plot, whereas downregulated genes such as PEDS1-UBE2V1 appear on the left. The corresponding heatmap (**Figure 2B**) further demonstrates clear segregation between PsA and PSO samples, reflecting distinct transcriptional signatures.

GO enrichment analysis (**Figure 2C**) revealed significant enrichment of biological processes related to extracellular region activity, platelet α-granules, plasminogen activators, fibrinogen complexes, coagulation and fibrin clot formation. These enriched terms point to enhanced vascular, endothelial, and hemostatic activity in PsA, distinguishing it from skin-limited psoriasis.

KEGG pathway analysis (**Figure 2D**) identified differential enrichment in multiple metabolic pathways, including ascorbate and aldarate metabolism, pentose and glucuronate interconversions, ether lipid metabolism, and cysteine and methionine metabolism. Notably, the complement and coagulation cascade was markedly upregulated in PsA, further supporting a shift toward endothelial and hemostatic activation.

Collectively, these findings highlight systemic immune and vascular activation in PsA that extends beyond cutaneous inflammation. The transcriptional divergence from PSO reinforces PsA as a distinct clinical and immunopathological entity rather than a mere extracutaneous manifestation of psoriasis.

### 3.4 Active vs remission PsA: activity-associated molecular signatures

**Figure 3:**
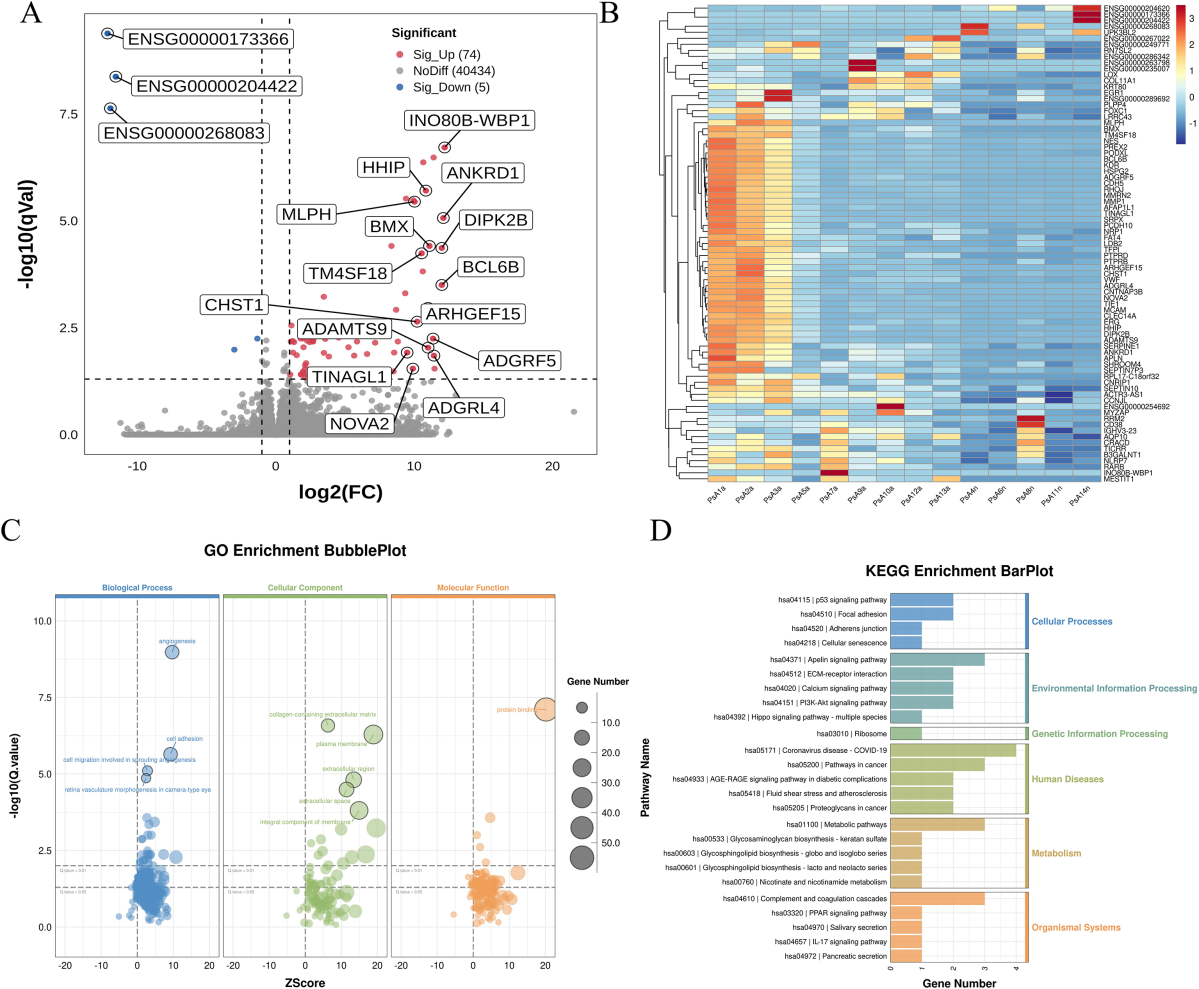
Transcriptomic landscape and pathway alterations associated with PsA disease activity (PsAa *vs* PsAn). **(A)** Volcano plot showing DEGs between active psoriatic arthritis (PsAa) and remission (PsAn) states. Significantly upregulated (red) and downregulated (blue) genes are shown based on thresholds of |log_2_FC| > 1 and FDR < 0.05. Representative DEGs are labeled. **(B)** Heatmap showing the expression patterns of DEGs across PsAa and PsAn samples. Rows represent genes and columns represent samples, with expression values displayed as z-scores (red: higher; blue: lower). **(C)** GO enrichment analysis of DEGs visualized by bubble plot across Biological Process, Cellular Component, and Molecular Function categories. **(D)** KEGG pathway enrichment analysis showing functional pathway categories, including upregulated pathways such as PI3K–Akt signaling, IL-17 signaling, Hippo signaling, and PPAR signaling in PsAa relative to PsAn. GO terms and KEGG pathways shown were selected based on nominal *p*-value < 0.05, with FDR values calculated using the Benjamini–Hochberg method. PBMCs, peripheral blood mononuclear cells; PsA, psoriatic arthritis; PsO, psoriasis; PsAa, active psoriatic arthritis; PsAn, psoriatic arthritis in remission states; DEGs, differentially expressed genes; GO, Gene Ontology; KEGG, Kyoto Encyclopedia of Genes and Genomes.

To elucidate transcriptional differences associated with disease activity in PsA, we compared PBMC transcriptomes from patients with active phase (PsAa, n = 9) and those in clinical remission (PsAn, n = 5). Differential expression analysis identified 74 significantly upregulated and 5 downregulated genes in the PsAa group (**Figure 3A**, **Supplementary Table 10**). Prominent upregulated transcripts—such as INO80B-WBP1, HHIP, ANKRD1, MLPH, BMX, DIPK2B, BCL6B, TM4SF18, CHST1, ARHGEF15, ADAMTS9, ADGRL4, ADGRF5, TINAGL1 and NOVA2—are involved in vascular biology, cellular signaling, and immune regulation. The 5 downregulated transcripts—among which ENSG0000173366, ENSG00000204422 and ENSG00000268083 showed the most pronounced suppression—may reflect impaired transcriptional or post-transcriptional control in the quiescence state. The heatmap (**Figure 3B**) demonstrates activity-related segregation of PsAa and PsAn samples based on their transcriptional profiles.

GO enrichment analysis (**Figure 3C**) revealed robust activation of angiogenesis, cell adhesion, and migration-related biological processes in PsAa. In the Cellular Component domain, DEGs were enriched in the plasma membrane, cell surface, and extracellular region, consistent with increased inflammatory trafficking. Protein binding emerged as the dominant Molecular Function category, reflecting broad engagement of receptor-ligand and signaling interactions.

KEGG pathway analysis (**Figure 3D**) revealed coordinated upregulation of multiple proinflammatory and immunoregulatory pathways, including PI3K-Akt, Hippo, IL-17, PPAR signaling, and the complement and coagulation cascades. In parallel, extensive metabolic rewiring was observed, involving glycosaminoglycan biosynthesis (keratan sulfate), glycosphingolipid biosynthesis, and nicotinate and nicotinamide metabolism—indicating that immune activation in PsA flares is tightly coupled with altered bioenergetic and biosynthetic programs.

**Figure 4:**
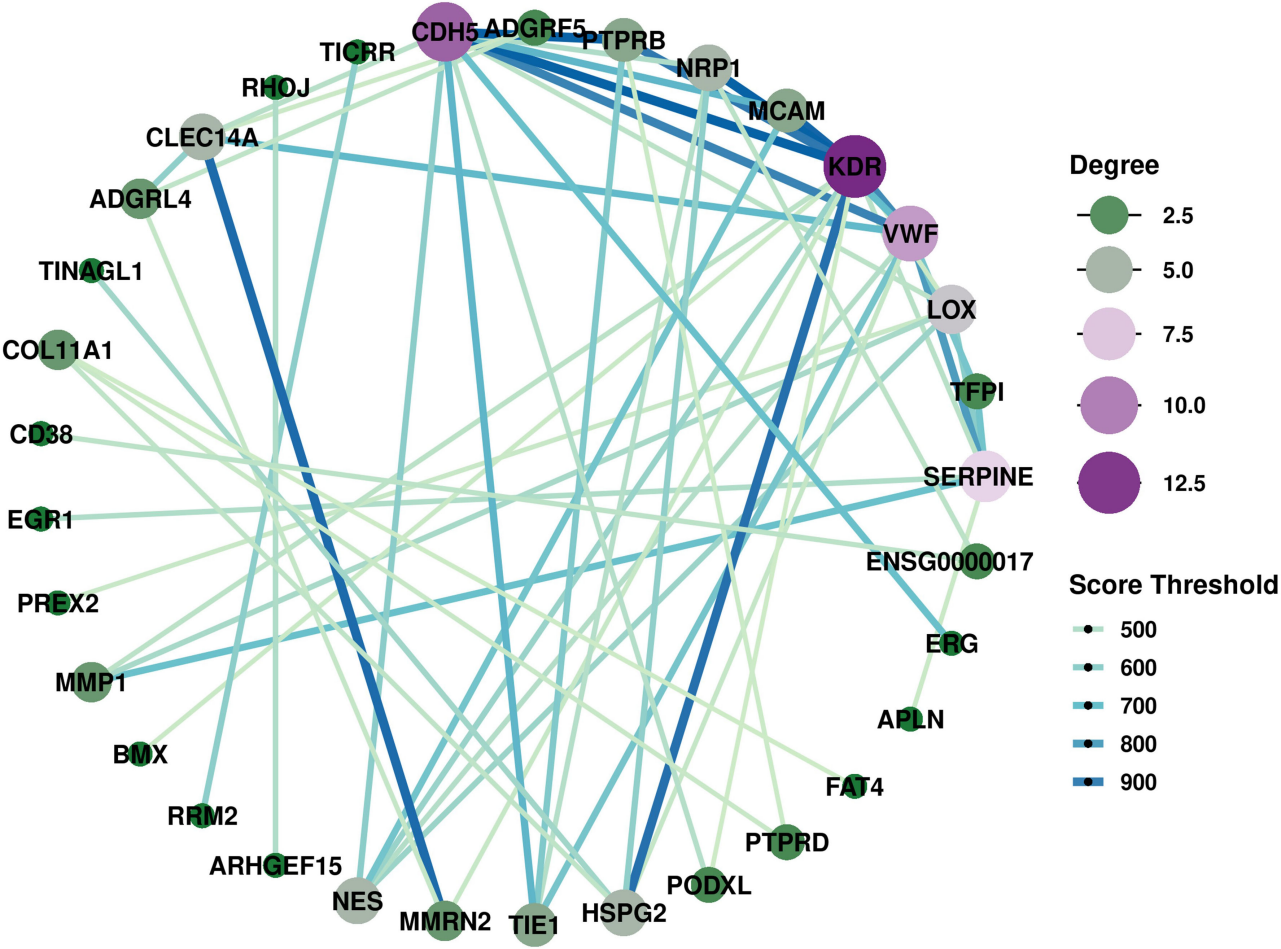
The PPI networks of DEGs between PsAa and PsAn was constructed using STRING (score > 0.4) and visualized in Cytoscape. Node size corresponds to degree centrality. CDH5, KDR, VWF, and SERPINE1 emerged as major hub genes within the network (see source data). PPI, Protein-protein interaction; PsAa, active psoriatic arthritis; PsAn, psoriatic arthritis in remission states; DEGs, differentially expressed genes.

To further characterize activity-associated interaction networks, we performed PPI analysis (**Figure 4**, **Supplementary Table 11**). Key hub nodes included CDH5, KDR, VWF, and SERPINE1, highlighted marked endothelial activation and angiogenic signaling, consistent with the well-established roles of neovascularization in PsA synovitis and enthesitis. GSEA (**Supplementary Figure 5**) supported these findings, highlighting upregulation of pathways related to protein adaptor activity, adenylate cyclase activity, and phosphatase activity, as well as biological processes such as blood vessel morphogenesis and monocyte differentiation.

Collectively, these transcriptomic alterations depict a coordinated immunometabolic activation state during PsA flare, characterized by heightened inflammatory signaling, vascular perturbation, and metabolic remodeling—providing mechanistic insights into disease flares and a framework for future biomarker development.

### 3.5 Pre- vs post-treatment: transcriptomic reversal and pathway suppression

**Figure 5:**
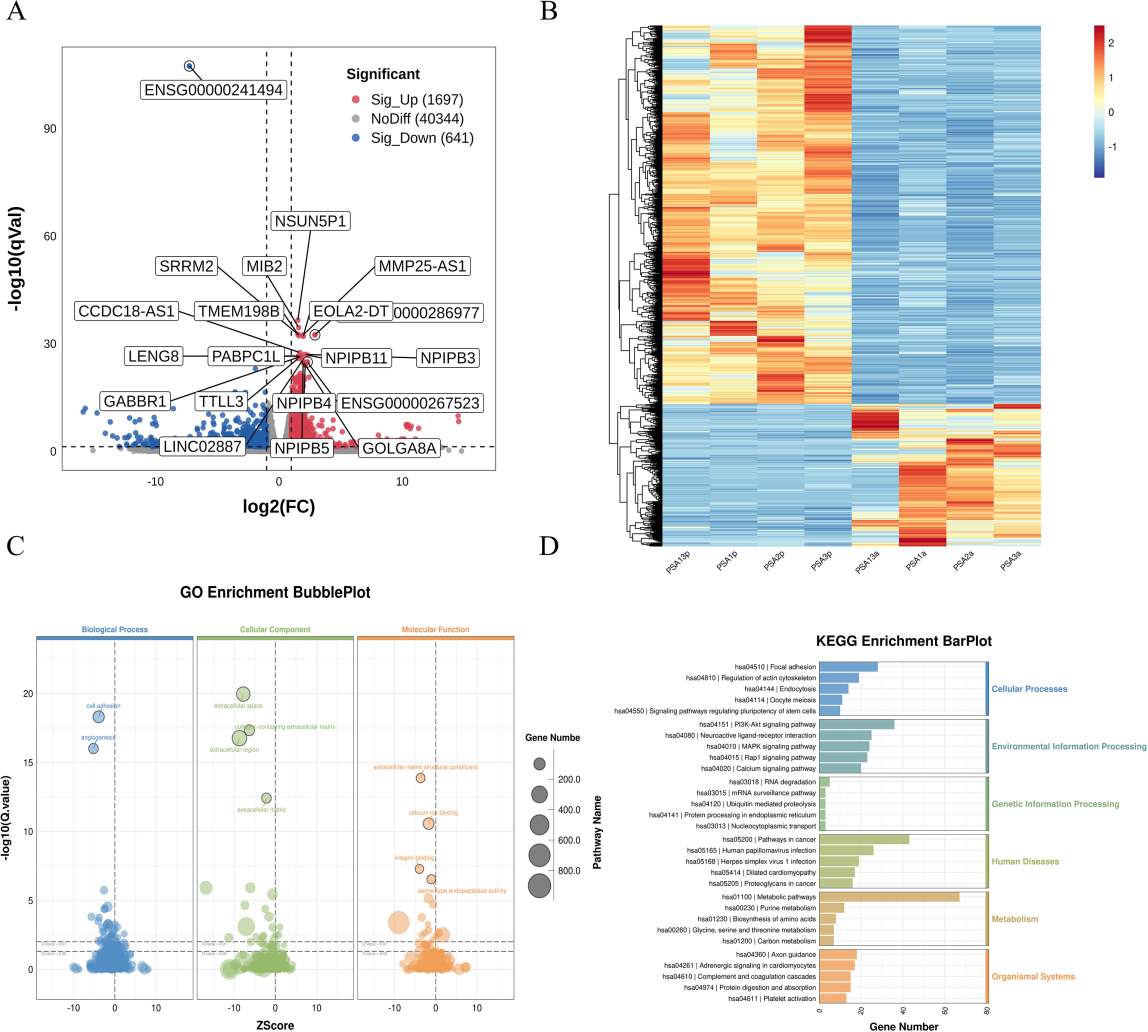
Transcriptomic alterations in PBMCs from PsA patients before and after treatment. **(A)** Volcano plot illustrating differentially expressed genes (DEGs) between baseline (day 0, PsAa) and post-treatment (day 90, PsAp) PBMCs. Red dots indicate significantly upregulated genes (|log_2_FC| > 1, FDR < 0.05), blue dots indicate downregulated genes, and grey dots represent non-significant genes. Representative DEGs are labeled. **(B)** Heatmap displaying the expression profiles of all DEGs across paired PsAa and PsAp samples. Rows denote genes and columns denote individual samples. Colors indicate normalized expression (z-score). **(C)** GO enrichment analysis of treatment-responsive DEGs across three categories: Biological Process (blue), Cellular Component (green), and Molecular Function (orange). The top enriched terms are displayed for each category. **(H)** KEGG pathway enrichment analysis of treatment-responsive DEGs across major functional domains, including cellular processes, environmental information processing, genetic information processing, metabolism, human diseases, and organismal systems. The most significantly enriched pathways are shown. GO terms and KEGG pathways shown were selected based on nominal *p*-value < 0.05, with FDR values calculated using the Benjamini–Hochberg method. PsA, psoriatic arthritis; PBMCs, peripheral blood mononuclear cells; PSAa, PsA patients before treatment; PSAp, PsA patients after treatment; GO, Gene Ontology; KEGG, Kyoto Encyclopedia of Genes and Genomes.

Paired transcriptomic profiling of PBMCs from 4 PsA patients before and after treatment (**Supplementary Table 12**) revealed 1,697 upregulated and 641 downregulated genes following therapy (**Supplementary Table 13**). The volcano plot and heatmap (**Figures 5A, B**) demonstrated clear segregation of pre- and post-treatment samples, indicating broad transcriptomic response, while volcano plots illustrated the distribution and magnitude of treatment-responsive genes. GO enrichment analysis (**Figure 5C**) revealed marked downregulation of biological processes related to angiogenesis and cell adhesion after treatment. In the Cellular Component category, suppressed genes were enriched in the extracellular space, extracellular matrix, and cell regions. In the Molecular Function domain, downregulated terms included extracellular matrix structural constituents, calcium ion binding, integrin binding, and serine-type endopeptidase activity—suggesting a reduction in inflammatory extracellular remodeling and diminished vascular reactivity following therapy.

KEGG pathway analysis (**Figure 5D**) showed consistent downregulation of several pathways implicated in PsA pathobiology. Key suppressed pathways included PI3K-Akt, Rap1, and coagulation cascades, as well as focal adhesion, extracellular matrix organization, and cell-surface receptor interactions. Together, these results indicate that treatment exerts broad immunomodulatory effects in PsA, dampening inflammatory signaling while simultaneously reducing pathological angiogenesis, extracellular matrix remodeling, and vascular–stromal interactions.

**Figure 6:**
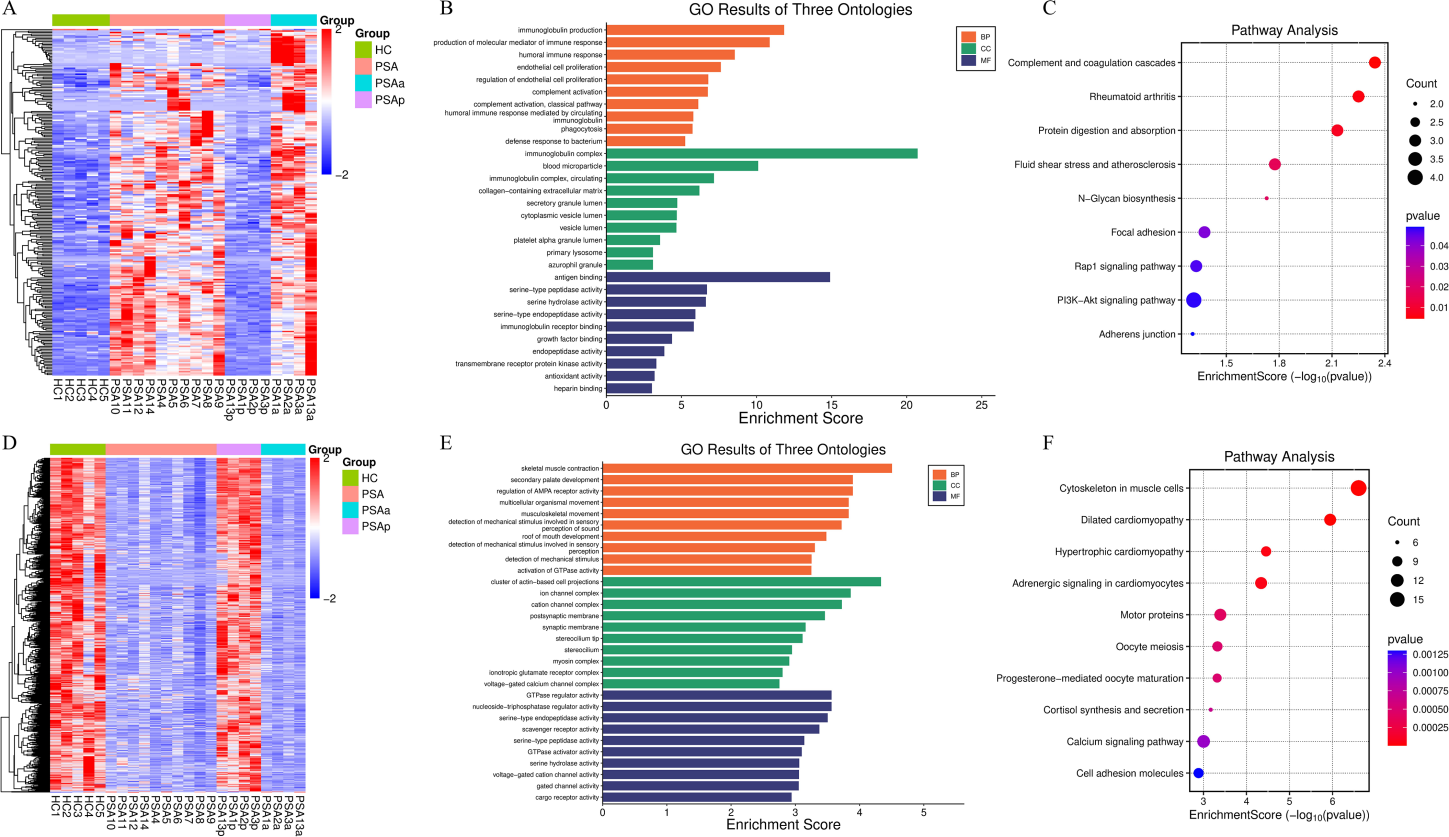
Reversal of PsA-associated transcriptional alterations following treatment. DEGs were first identified by comparing PsA patients with HC, and subsequently examined for treatment-associated expression changes between PsAa and PsAp samples. **(A)** Heatmap showing DEGs that were upregulated in PsA compared with HC and subsequently downregulated after treatment (PsAp *vs* PsAa). Rows represent genes and columns represent individual samples. Colors indicate normalized expression levels. **(B)** GO enrichment analysis of the genes shown in panel A, including Biological Process, Cellular Component, and Molecular Function categories. **(C)** KEGG pathway enrichment dot plot of the same gene set shown in panel **(A)** Dot size represents the number of genes mapped to each pathway, and color indicates enrichment significance. **(D)** Heatmap showing DEGs that were downregulated in PsA compared with HC and subsequently upregulated after treatment (PsAp *vs* PsAa). **(E)** GO enrichment analysis of the genes shown in panel **(D, F)** KEGG pathway enrichment dot plot of the same gene set shown in panel **(D)** Together, these analyses highlight PsA-associated transcriptional programs that exhibit partial normalization following treatment, involving immune-related, metabolic, and signaling pathways. PsA, psoriatic arthritis; HC, healthy controls; DEGs, differentially expressed genes; PSAa, PsA patients before treatment; PSAp, PsA patients after treatment; GO, Gene Ontology; BP, Biological Process; CC, Cellular Component; MF, Molecular Function; KEGG, Kyoto Encyclopedia of Genes and Genomes.

To further characterize treatment-associated transcriptional changes, we examined PsA–HC DEGs that exhibited expression reversal following therapy. Among all DEGs, 1,352 genes (**Supplementary Table 14**) showed reversal toward the baseline (healthy-like) direction after treatment, including 233 genes that were upregulated in PsA and subsequently downregulated after treatment (**Figure 6A**), and 1,119 genes that were downregulated in PsA and showed increased expression post-treatment (**Figure 6D**). Functional enrichment analysis revealed that genes upregulated in PsA and downregulated after treatment were primarily enriched in immune-related processes, including complement activation, humoral immune responses, and inflammatory signaling pathways (**Figures 6B, C**). In contrast, genes downregulated in PsA and restored after treatment were enriched in pathways related to cytoskeletal organization, cell adhesion, and calcium signaling, suggesting partial normalization of cellular structural and signaling programs following therapy (**Figures 6E, F**).

**Figure 7:**
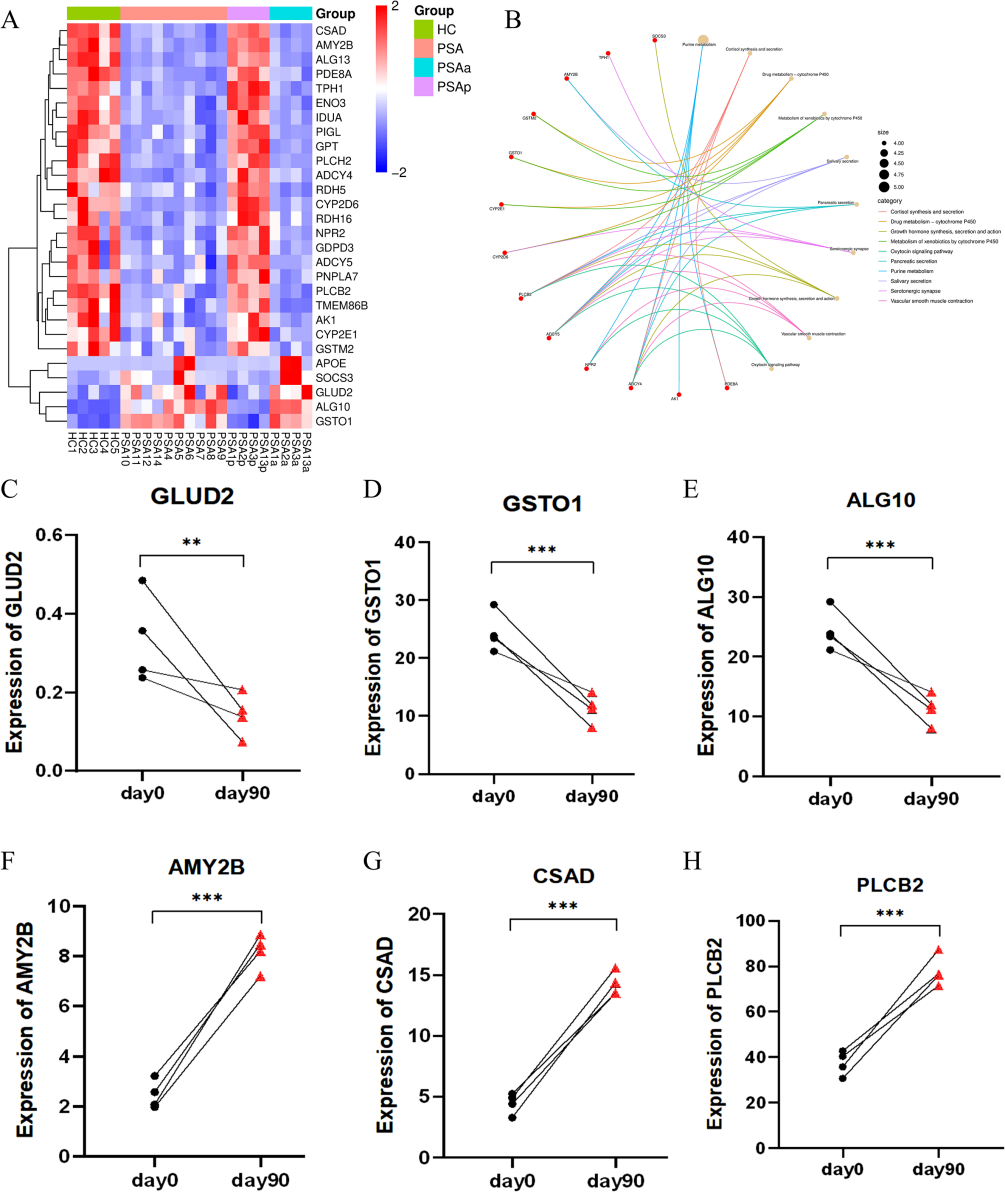
Transcriptomic reversal of PsA-associated metabolic genes following treatment. **(A)** Heatmap showing the expression profiles of 28 metabolism-related DEGs that exhibited treatment-associated reversal toward HC-like expression patterns. These genes were identified based on KEGG metabolic pathway annotation and displayed expression changes from PsAa to PsAp that were directionally opposite to the baseline PsA–HC differences. **(B)** KEGG pathway enrichment network of metabolism-related DEGs showing their association with key metabolic and signaling pathways. Nodes represent genes or pathways, and edges indicate gene–pathway relationships. **(C-H)** Paired expression changes of six metabolic-related DEGs in PBMCs from four PsA patients before treatment (PSAa, day 0; black dots) and after treatment (PSAp, day 90; red triangles). GLUD2, GSTO1, and ALG10 showed significant post-treatment downregulation, while AMY2B, CSAD, and PLCB2 exhibited significant upregulation (*P*<0.05 *, *P*<0.01 **, *P*<0.001 ***, paired t-test or Wilcoxon matched-pairs test). PsA, psoriatic arthritis; HC, healthy controls; DEGs, differentially expressed genes; PSAa, PsA patients before treatment; PSAp, PsA patients after treatment;KEGG, Kyoto Encyclopedia of Genes and Genomes.

Given the high prevalence of metabolic comorbidities in PsA, we next focused on metabolic genes within the set of treatment-associated reversal DEGs. Specifically, metabolism-related genes were directly selected from the reversal DEG set based on KEGG metabolic pathway annotation. Of these, 28 metabolism-related genes exhibited treatment-associated reversal trends, defined as expression changes from pre- to post-treatment that were directionally opposite to the baseline differences observed between PsA and HC (**Supplementary Table 15**). Specifically, genes that were downregulated in PsA compared with HC showed increased expression after treatment, whereas genes upregulated at baseline demonstrated decreased expression post-treatment, with expression levels shifting toward those observed in HC (**Figure 7A**). Of the 28 metabolism-related genes showing reversal trends (**Figure 7B**), 23 were upregulated after treatment, including AMY2B, CSAD, and PLCB2, while 5 genes were downregulated, such as GLUD2, GSTO1, and ALG10 (**Figures 7C–H**). Notably, this analysis was intended to describe treatment-associated directional shifts in gene expression rather than formal statistical normalization to HC levels, highlighting partial restoration of metabolic transcriptional programs.

## 4 Discussion

This study integrates four complementary transcriptomic comparisons—PsA versus healthy controls, PsA versus psoriasis-only, active versus remission PsA, and paired pre- versus post-treatment samples—to delineate systemic immune alterations across disease states. This multi-layered approach provides a structured overview of immune, vascular, and metabolic programs associated with PsA.

Compared with healthy individuals, PsA PBMCs exhibited extensive transcriptional alterations, with prominent activation of adaptive immune responses, antigen presentation, and T cell–mediated pathways. These findings align with the established immunologic features of PsA (21–23) and highlight the contribution of peripheral immune cells to its immunopathology (24–26). Concurrent enrichment of oxidative phosphorylation, fatty acid utilization, and amino acid metabolism suggests that these immune responses are accompanied by substantial metabolic adjustment supporting heightened cellular activation (27–29). Notably, the differential expression of LY96 (30), S100A4 (31), and CAPG (32)—identified from the transcriptomic analysis, shared across available external datasets, and implicated in PsA-associated immune activation—was independently confirmed by qRT-PCR. This independent validation underscores the robustness of our transcriptomic findings and supports their biological relevance in PsA. The comparison between PsA and psoriasis-only revealed a distinct pattern of systemic involvement. Genes related to extracellular matrix organization, platelet activation, fibrinolysis, coagulation, and complement pathways were more pronounced in PsA, underscoring the presence of vascular and hemostatic alterations that are less evident in skin-limited disease. Differences in cholesterol and aldehyde metabolism further suggest a distinct metabolic milieu in PsA. These results reinforce that PsA is biologically distinct from psoriasis alone, with broader systemic inflammatory and vascular features (33). Disease activity was associated with an additional layer of transcriptional alteration. Active PsA demonstrated increased expression of genes involved in angiogenesis, cell adhesion, leukocyte trafficking, and extracellular matrix remodeling. Enrichment of PI3K-Akt, MAPK, IL-17, and complement/coagulation pathways reflects the convergence of inflammatory and stromal activation during active disease (34). Altered glycosaminoglycan, lipid, and redox metabolism further illustrates the metabolic component of inflammatory amplification. Longitudinal profiling revealed substantial transcriptomic reversibility following treatment. Many activity-associated genes shifted toward baseline expression, accompanied by attenuation of angiogenesis, extracellular matrix remodeling, complement/coagulation cascades, and inflammatory signaling (35, 36). In addition, several genes involved in metabolic regulation and intracellular signaling exhibited treatment-associated transcriptional changes, with GLUD2, GSTO1, and ALG10 significantly downregulated and AMY2B, CSAD, and PLCB2 upregulated after therapy. These genes encode proteins previously implicated in immune metabolism, redox balance, and receptor-mediated signaling. Although protein-level validation was not performed in this study, the observed mRNA changes are directionally consistent with existing protein-level and functional evidence in inflammatory contexts (37–43), supporting their biological relevance. Nevertheless, further studies are required to determine how these transcriptional shifts relate to clinical improvement and whether they are influenced by specific therapeutic agents.

Taken together, the four comparisons reveal several consistent themes: 1.Peripheral immune activation is a consistent feature of PsA across clinical states. 2. Vascular and coagulation pathways emerge prominently when distinguishing PsA from PsO, suggesting systemic involvement that extends beyond skin inflammation. 3. Metabolic remodeling appears across multiple comparisons, indicating a sustained shift in immunometabolic states in PsA. 4. Treatment partially reverses these alterations, underscoring the dynamic nature of peripheral immune signatures. Rather than implying diagnostic or biomarker utility, these observations provide a framework for understanding systemic inflammatory and metabolic characteristics in PsA at the transcriptomic level.

Compared to prior transcriptomic studies in PsA—many of which have focused on isolated tissue compartments such as skin (44) or synovium (45) or examined PBMCs at a single disease state—our analysis provides a more integrated view of systemic immune alterations across multiple clinical contexts. Recent multi-omics investigations, including those by Groen et al. (26) and Li et al. (34) have highlighted protease- and miRNA-mediated pathways in PsA, suggesting complex regulatory mechanisms underlying disease fluctuations. However, transcriptomic characterizations capturing both disease activity and treatment-related shifts remain limited. By combining RNA-seq with detailed clinical phenotyping, the present study delineates activity-related and treatment-responsive transcriptional patterns, particularly within metabolic and matrix-{{-}}-associated pathways. In addition, the direct comparison with cutaneous PsO helps clarify transcriptional features that appear more specific to PsA, including coagulation and vascular signatures. These observations complement existing datasets and contribute to a more comprehensive understanding of systemic inflammatory states in PsA.

### 4.1 Limitations

This study has several limitations. The sample size, particularly for psoriasis-only and healthy control groups, was modest, which may limit statistical power and generalizability. Although publicly available PBMC transcriptomic datasets were explored, disease heterogeneity and differences in data processing resulted in limited gene overlap, precluding robust cross-dataset validation; thus, this work should be interpreted as exploratory. Not all analyses were adjusted for potential confounders; only comparisons between PsA and healthy controls or psoriasis included age, sex, and BMI. Consequently, some observed transcriptomic differences or pathway alterations could partially reflect unaccounted demographic or metabolic factors rather than disease-specific effects. Transcript-level alterations were not confirmed at the protein or functional level, and PBMCs may not fully capture synovial or entheseal processes central to PsA. Treatment heterogeneity may influence longitudinal observations. Given the high prevalence of metabolic comorbidities in PsA, KEGG-annotated metabolism-related genes showing treatment-associated reversal trends were examined; however, further metabolomic or functional studies are warranted. Collectively, these limitations should be considered when interpreting the findings and planning future studies.

### 4.2 Conclusion

In summary, this multi-dimensional transcriptomic analysis provides an integrated view of systemic immune and metabolic alterations in PsA across health, disease activity, and treatment states. The results enhance the understanding of circulating inflammatory patterns in PsA and offer a foundation for future mechanistic and translational studies.

## Data Availability

The datasets presented in this study can be found in online repositories. The names of the repository/repositories and accession number(s) can be found in the article/**Supplementary Material**.

## Supplementary material

Supplementary Figure 1 PCA of normalized PBMC transcriptomic profiles across all participant groups. PCA was performed on the voom-transformed expression matrix of 42,682 genes retained after quality control. Each point represents an individual sample. **(A)** PCA comparing PsA patients and HC, showing distinct separation along PC1 and PC2. **(B)** PCA of PsO vs HC samples demonstrating clear group-level divergence. **(C)** PCA comparing PsA and PsO groups, illustrating partial overlap but overall separation, indicating both shared and distinct systemic immune signatures. **(D)** PCA of active PsA (PsAa) and remission PsA (PsAn), showing partially overlapping but distinguishable clusters corresponding to disease activity states. **(E)** Paired PCA of PsA patients before treatment (PsAa) and after treatment (PsAp), demonstrating consistent within-patient shifts in global expression profiles following therapy. No outliers were detected. PCA, principal component analysis; PsA, psoriatic arthritis; PsO, psoriasis; HC, healthy controls; PsAa, active psoriatic arthritis; PsAn, psoriatic arthritis in remission states; paired PSAa, PsA patients before treatment; PSAp, PsA patients after treatment.

Supplementary Figure 2 GSEA of PBMC transcriptomes from PsA patients versus HC. GSEA revealed broad activation of immune and inflammatory pathways in PsA. Upregulated pathways include T cell receptor signaling, Th17 cell differentiation, NF-κB signaling, cytokine-cytokine receptor interaction, VEGF signaling, Fcγ receptor-mediated phagocytosis, PI3K-Akt signaling, and MAPK signaling. Multiple metabolic pathways (e.g., arginine and proline metabolism, unsaturated fatty acid biosynthesis, cysteine and methionine metabolism) and immune-related pathways (MAPK, p53, Rap1, complement and coagulation cascades) were significantly upregulated in PsA compared to controls. Gene sets were considered significant if they met the criteria of |NES| > 1, nominal *p*-value < 0.05, and FDR q-value < 0.25. PBMCs, peripheral blood mononuclear cells; PsA, psoriatic arthritis; HC, healthy controls; GSEA, gene set enrichment analysis.

Supplementary Figure 3 Limited overlap between PsA *versus* HC DEGs and publicly available PBMC transcriptomic datasets. Venn diagram showing the overlap of DEGs identified in our PsA *versus* HC comparison with two publicly available PBMC transcriptomic datasets (GSE179800 and GSE137510). Although each dataset contained a substantial number of expressed genes, only eight genes were consistently shared across all three datasets. This limited overlap likely reflects differences in cohort composition, disease heterogeneity, and data processing strategies, including pre-filtering of low-abundance transcripts in public datasets. PBMCs, peripheral blood mononuclear cells; PsA, psoriatic arthritis; HC, healthy controls;DEGs, differentially expressed genes.

Supplementary Figure 4 Validation of selected differentially expressed genes by qRT-PCR. Quantitative real-time PCR analysis of **(A)** LY96, **(B)** S100A4, and **(C)** CAPG expression in PBMCs from patients with PsA and HC. Gene expression levels were normalized to GAPDH and calculated using the 2^−ΔΔCt method. Data are presented as mean ± standard deviation (SD). *P* < 0.05 was considered statistically significant. PBMCs, peripheral blood mononuclear cells; PsA, psoriatic arthritis; HC, healthy controls.

Supplementary Figure 5 GSEA plots of gene sets significantly enriched in PsAa relative to PsAn. Enriched molecular function categories included protein macromolecule adaptor activity, activation of adenylate cyclase activity, and phosphatase activity. Enriched biological processes and pathways included signaling receptor activity, adenylate cyclase–inhibiting G protein–coupled receptor signaling, blood vessel morphogenesis, cellular response to VEGF stimulus, carbohydrate binding, and monocyte differentiation. Gene sets were considered significant if they met the criteria of |NES| > 1, nominal *p*-value < 0.05, and FDR q-value < 0.25. PsAa, active psoriatic arthritis; PsAn, psoriatic arthritis in remission states; GSEA, gene set enrichment analysis.

Supplementary Method 1 Pathway Enrichment and Protein–Protein Interaction (PPI) Network Analysis.

Supplementary Table 1 Differentially expressed genes between PsA patients and healthy controls.

Supplementary Table 2 Gene symbols corresponding to the Venn overlap identified in GSE137510.

Supplementary Table 3 Gene symbols corresponding to the Venn overlap identified in GSE179800.

Supplementary Table 4 Gene symbols from GSE137510 corresponding to the Venn overlap with GSE179800.

Supplementary Table 5 Gene symbols corresponding to the Venn overlap in PsA versus healthy controls.

Supplementary Table 6 Gene symbols from GSE137510 corresponding to the Venn overlap with PsA *versus* healthy controls.

Supplementary Table 7 Gene symbols from GSE179800 corresponding to the Venn overlap with PsA versus healthy controls.

Supplementary Table 8 Gene symbols corresponding to the Venn overlap of PsA *versus* healthy control differentially expressed genes across GSE179800 and GSE137510.

Supplementary Table 9 Differentially expressed genes between PsA and PsO patients.

Supplementary Table 10 Differentially expressed genes between active and remission PsA patients.

Supplementary Table 11 Protein_Interaction between active and remission PsA patients.

Supplementary Table 12 Clinical characteristics of four PsA patients with paired pre- and post-treatment transcriptomic data.

Supplementary Table 13 Differentially expressed genes before and after treatment in PsA patients.

Supplementary Table 14 Differentially expressed genes reversed after treatment in PsA patients.

Supplementary Table 15 Treatment-Reversed Metabolism-Related Differentially Expressed Genes in PsA.
