# Supplementary figures and images for: PBMC transcriptomic signatures reflect immune dynamics and disease activity in psoriatic arthritis
Source: Front Immunol. 2026 Feb 24;17:1701395. doi: 10.3389/fimmu.2026.1701395 (PMC12971704; doi:10.3389/fimmu.2026.1701395)

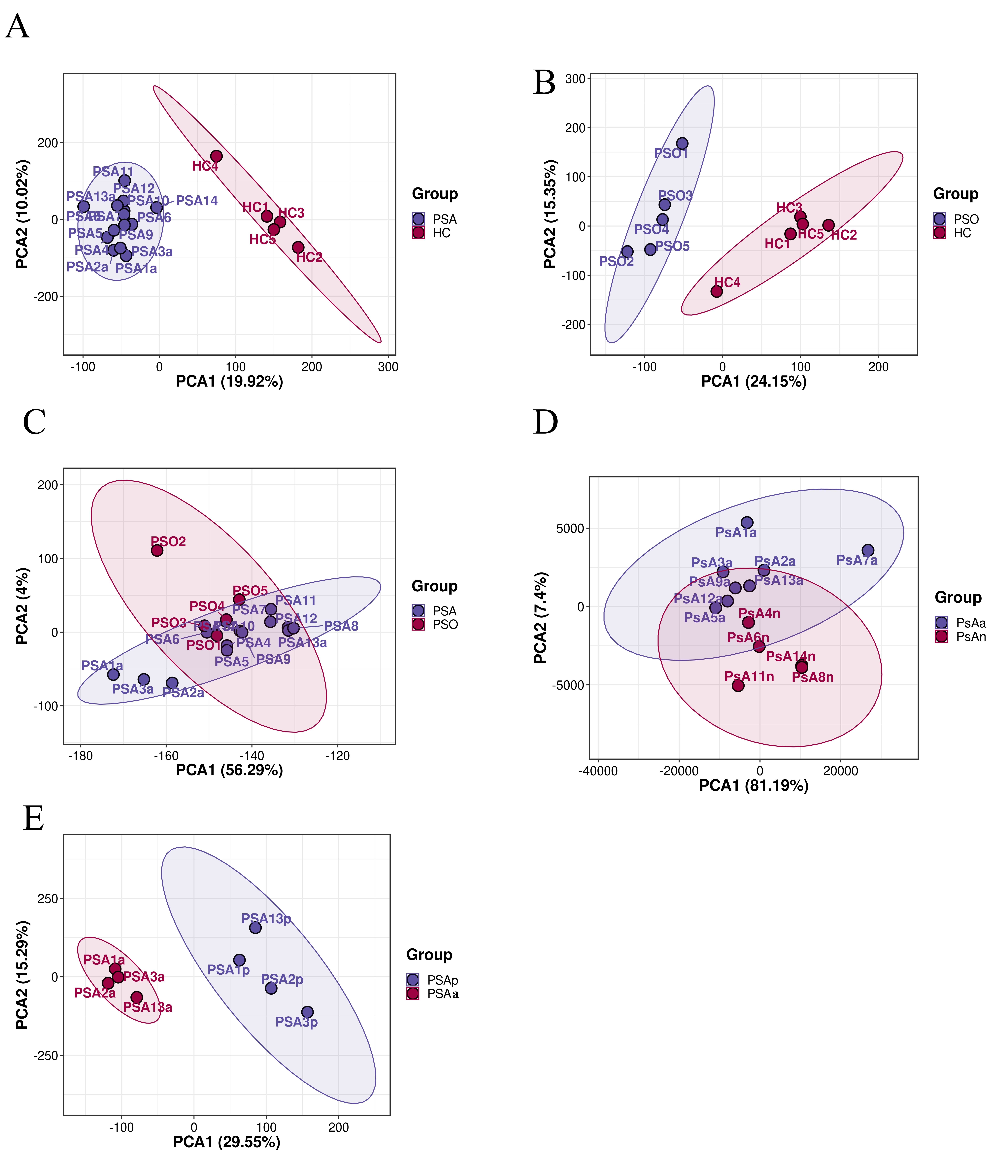

Supplement: Supplementary Figure 1 — PCA of normalized PBMC transcriptomic profiles across all participant groups. PCA was performed on the voom-transformed expression matrix of 42,682 genes retained after quality control. Each point represents an individual sample. (A) PCA comparing PsA patients and HC, showing distinct separation along PC1 and PC2. (B) PCA of PsO vs HC samples demonstrating clear group-level divergence. (C) PCA comparing PsA and PsO groups, illustrating partial overlap but overall separation, indicating both shared and distinct systemic immune signatures. (D) PCA of active PsA (PsAa) and remission PsA (PsAn), showing partially overlapping but distinguishable clusters corresponding to disease activity states. (E) Paired PCA of PsA patients before treatment (PsAa) and after treatment (PsAp), demonstrating consistent within-patient shifts in global expression profiles following therapy. No outliers were detected. PCA, principal component analysis; PsA, psoriatic arthritis; PsO, psoriasis; HC, healthy controls; PsAa, active psoriatic arthritis; PsAn, psoriatic arthritis in remission states; paired PSAa, PsA patients before treatment; PSAp, PsA patients after treatment. [file Image1.jpeg]

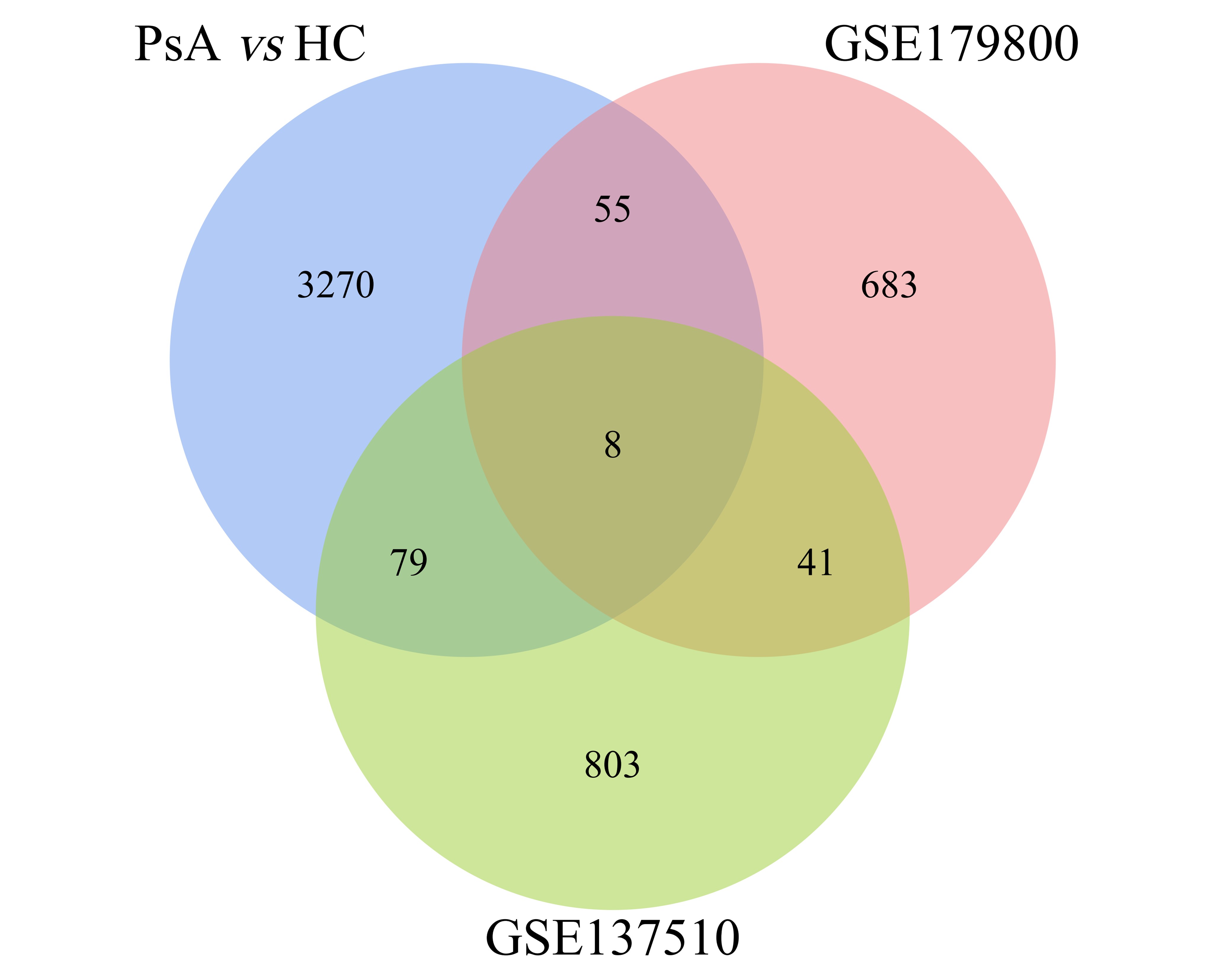

Supplement: Supplementary Figure 3 — Limited overlap between PsA versus HC DEGs and publicly available PBMC transcriptomic datasets. Venn diagram showing the overlap of DEGs identified in our PsA versus HC comparison with two publicly available PBMC transcriptomic datasets (GSE179800 and GSE137510). Although each dataset contained a substantial number of expressed genes, only eight genes were consistently shared across all three datasets. This limited overlap likely reflects differences in cohort composition, disease heterogeneity, and data processing strategies, including pre-filtering of low-abundance transcripts in public datasets. PBMCs, peripheral blood mononuclear cells; PsA, psoriatic arthritis; HC, healthy controls;DEGs, differentially expressed genes. [file Image3.jpeg]

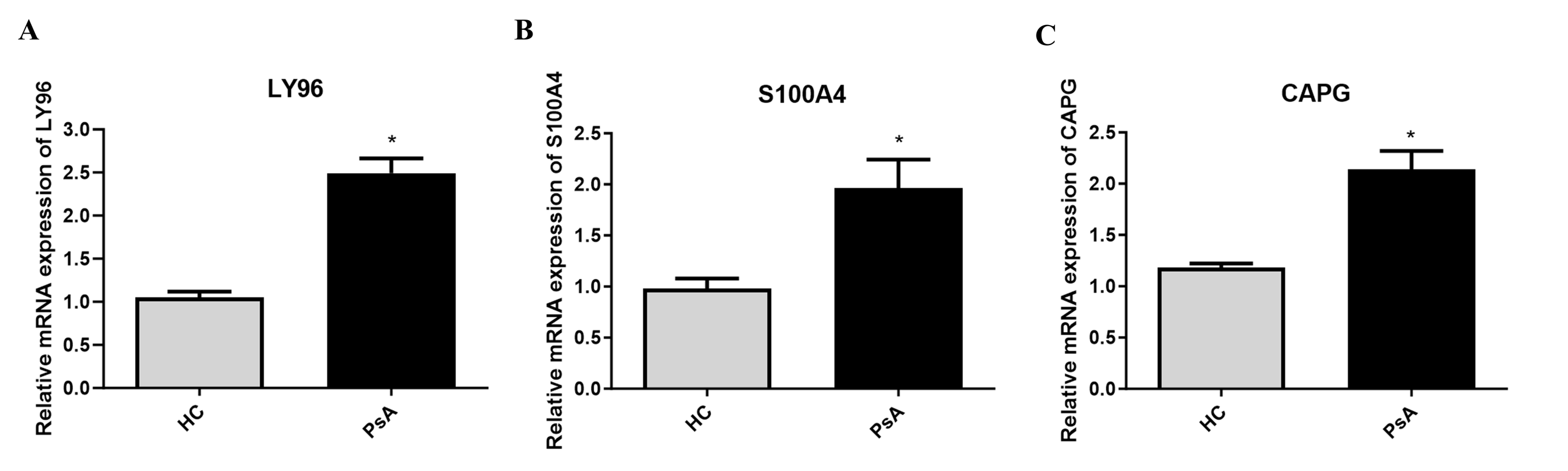

Supplement: Supplementary Figure 4 — Validation of selected differentially expressed genes by qRT-PCR. Quantitative real-time PCR analysis of (A) LY96, (B) S100A4, and (C) CAPG expression in PBMCs from patients with PsA and HC. Gene expression levels were normalized to GAPDH and calculated using the 2^−ΔΔCt method. Data are presented as mean ± standard deviation (SD). P < 0.05 was considered statistically significant. PBMCs, peripheral blood mononuclear cells; PsA, psoriatic arthritis; HC, healthy controls. [file Image4.jpeg]

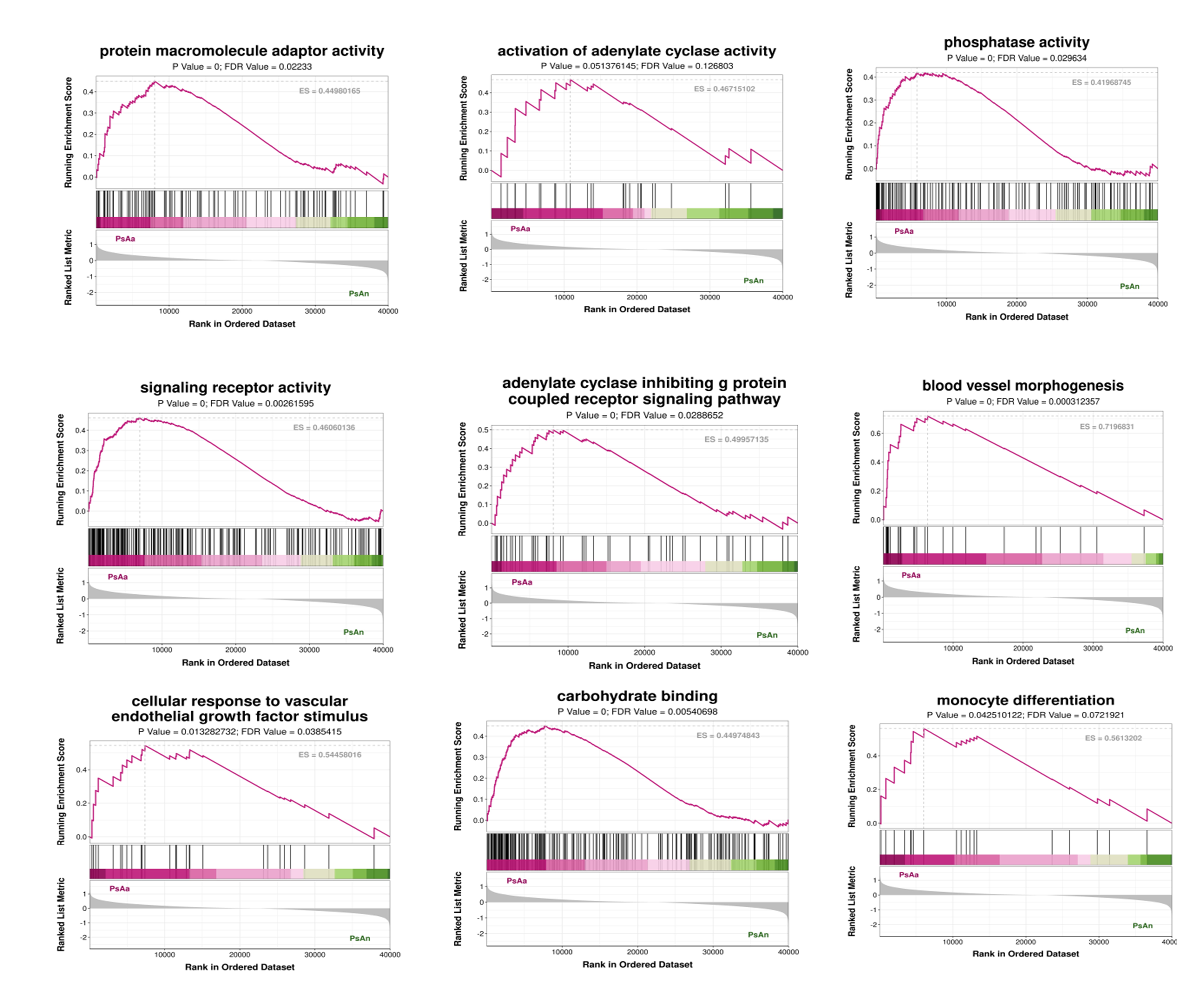

Supplement: Supplementary Figure 5 — GSEA plots of gene sets significantly enriched in PsAa relative to PsAn. Enriched molecular function categories included protein macromolecule adaptor activity, activation of adenylate cyclase activity, and phosphatase activity. Enriched biological processes and pathways included signaling receptor activity, adenylate cyclase–inhibiting G protein–coupled receptor signaling, blood vessel morphogenesis, cellular response to VEGF stimulus, carbohydrate binding, and monocyte differentiation. Gene sets were considered significant if they met the criteria of |NES| > 1, nominal p-value < 0.05, and FDR q-value < 0.25. PsAa, active psoriatic arthritis; PsAn, psoriatic arthritis in remission states; GSEA, gene set enrichment analysis. [file Image5.jpeg]
