# Supplementary material for: PBMC transcriptomic signatures reflect immune dynamics and disease activity in psoriatic arthritis
Source: Front Immunol. 2026 Feb 24;17:1701395. doi: 10.3389/fimmu.2026.1701395 (PMC12971704; doi:10.3389/fimmu.2026.1701395)
Supplement: Supplementary Method 1 — Pathway Enrichment and Protein–Protein Interaction (PPI) Network Analysis. [file DataSheet1.zip › Supplement Table 12 .docx]

Supplement Table 1: Clinical characteristics of four PsA patients with paired pre- and post-treatment transcriptomic data.

| Patient No. | Age, years | Sex | Treatment | Concomitant medications | Treatment  duration | DAPSA score | PASI score  pre- post- | CRP  pre- post- | Clinical response |
| --- | --- | --- | --- | --- | --- | --- | --- | --- | --- |
|  |  |  |  |  |  | pre- post- |  |  |  |
| PSA 1 | 41 | F | Adalimumab 80 mg loading → 40 mg q2w | NSAIDs | 3 months | 25.2 1.1 | 5.9 0.3 | 10 1 | Good |
| PSA 2 | 47 | M | Secukinumab 300 mg weekly ×5 → 300 mg monthly | NSAIDs | 3 months | 34 3.2 | 7.4 0.5 | 12 2 | Good |
| PSA 3 | 47 | M | Adalimumab 80 mg loading → 40 mg q2w | NSAIDs | 3 months | 48.82 3.6 | 5.3 0.9 | 18.2 6 | Good |
| PSA 13 | 40 | F | Secukinumab 300 mg weekly ×5 → 300 mg monthly | NSAIDs | 3 months | 40.80 4.3 | 2.2 0.8 | 38 3 | Good |

Abbreviations: DAPSA: disease activity index for psoriatic arthritis; PASI: psoriasis area and severity index; CRP: C-reactive protein.

Clinical response was defined as a ≥50% reduction in DAPSA score from baseline, consistent with established GRAPPA/EULAR recommendations.
