# Supplementary material for: PBMC transcriptomic signatures reflect immune dynamics and disease activity in psoriatic arthritis
Source: Front Immunol. 2026 Feb 24;17:1701395. doi: 10.3389/fimmu.2026.1701395 (PMC12971704; doi:10.3389/fimmu.2026.1701395)
Supplement: Supplementary Method 1 — Pathway Enrichment and Protein–Protein Interaction (PPI) Network Analysis. [file DataSheet1.zip › Supplementary Methodú║Pathway Enrichment and Protein-Protein Interaction Network Analysis.docx]

PPI network construction:

PPI networks were generated using the STRING database (v11.5). Only DEGs that passed our statistical thresholds (|log₂FC| > 2 and FDR < 0.05) were included. No first- or second-order neighbours were added, ensuring that the network reflects interactions specifically among the identified DEGs. Interactions with a confidence score > 0.4 (medium-to-high confidence) were retained.

Cytoscape analysis:

The STRING interaction tables were imported into Cytoscape (v3.9.1) for further network interrogation.

CytoHubba was used to identify hub genes based on the Maximal Clique Centrality (MCC) algorithm.

MCODE was applied to detect densely connected subnetworks or key interaction modules.
